# Secretory VSMC-derived THBS1 promotes macrophage M1 polarization and inflammation in intracranial aneurysms via the CD47/NF-κB axis

**DOI:** 10.3389/fimmu.2026.1790488

**Published:** 2026-04-10

**Authors:** Jianhuang Huang, Qixiu Wang, Yangyang Lin, Jianning Chen, Caihou Lin

**Affiliations:** 1 Department of Neurosurgery, Affiliated Hospital of Putian University, Putian, Fujian, China; 2 Department of Cerebral Diseases Rehabilitation III, Affiliated Hospital of Liaoning University of Traditional Chinese Medicine, Shenyang, Liaoning, China; 3 Department of Obstetrics and Gynecology, Chinese People’s Armed Police Force Fujian Provincial Corps Hospital, Fuzhou, Fujian, China; 4 Department of Neurosurgery, Fujian Medical University Union Hospital, Fuzhou, Fujian, China

**Keywords:** CD47, intercellular communication, intracranial aneurysm, M1 macrophage, thrombospondin-1, VSMC phenotypic switching

## Abstract

**Background:**

Chronic vascular inflammation and immune cell infiltration are key pathogenic features of intracranial aneurysms (IAs). Although phenotypic switching of vascular smooth muscle cells (VSMCs) and macrophage M1 polarization have both been observed in IAs, the intercellular communication mechanisms linking these two processes remain incompletely understood. This study aimed to explore the potential role of the THBS1–CD47 axis in mediating crosstalk between VSMCs and macrophages within the IA microenvironment.

**Methods:**

We analyzed bulk transcriptomic (GSE54083) and single-cell RNA-sequencing (GSE193533) datasets to characterize the cellular landscape of IAs. Weighted gene co-expression network analysis (WGCNA) and the CellChat algorithm were applied to predict key ligand–receptor interactions. To validate these predictions *in vitro*, human aortic VSMCs (HA-VSMCs) were induced toward a secretory phenotype and co-cultured with THP-1–derived macrophages. shRNA-mediated THBS1 knockdown, CD47-neutralizing antibodies, and NF-κB inhibitors were used to investigate the underlying mechanisms.

**Results:**

Bioinformatic analyses suggested enrichment of M1-like macrophages and phenotypically switched VSMCs in IA tissues compared with controls. CellChat analysis identified THBS1–CD47 as a potential key ligand–receptor pair linking secretory VSMCs and macrophages. Consistent with these predictions, *in vitro* experiments showed that secretory VSMCs released elevated levels of THBS1, which correlated with increased expression of M1 markers (CD86, iNOS) and pro-inflammatory cytokines in co-cultured macrophages. Mechanistic studies suggested that VSMC-derived THBS1 may promote this inflammatory response by activating the CD47/NF-κB signaling axis, as blockade of this pathway markedly attenuated macrophage M1 polarization.

**Conclusions:**

Collectively, our findings support a model in where secretory VSMCs may actively regulate the immune microenvironment of IAs through paracrine secretion of THBS1. This signaling appears to drive macrophage M1 polarization via the CD47/NF-κB axis. Targeting the THBS1–CD47 interaction may represent a promising therapeutic strategy to alleviate vascular inflammation in IAs.

## 1 Introduction

Intracranial aneurysm (IA) is a common cerebrovascular disorder with a prevalence of approximately 3–5% in the general population (1). Although most aneurysms remain asymptomatic, rupture leads to subarachnoid hemorrhage (SAH), a catastrophic event associated with high mortality and morbidity (2). At present, microsurgical clipping and endovascular embolization are the main therapeutic options; however, these invasive interventions carry inherent procedural risks and are not suitable for all patients. Therefore, elucidating the molecular mechanisms underlying IA formation and rupture and identifying pharmacological targets capable of stabilizing the aneurysmal wall are urgent needs in neurosurgery.

Pathologically, IAs are characterized by chronic inflammation of the vessel wall, extracellular matrix (ECM) degradation, and apoptosis or phenotypic switching of vascular smooth muscle cells (VSMCs) (3). Accumulating evidence suggests that the immune-inflammatory microenvironment plays a critical role in IA progression. In particular, macrophage infiltration and polarization status are considered key drivers of vascular wall destruction (4, 5). Macrophages exhibit high plasticity and can differentiate into pro-inflammatory M1 or anti-inflammatory/reparative M2 phenotypes. In IA lesions, M1 macrophages are markedly increased and accelerate vascular wall remodeling and aneurysm growth by secreting inflammatory cytokines such as interleukin-1β (IL-1β) and tumor necrosis factor-α (TNF-α), as well as matrix metalloproteinases (MMPs) (4, 6). However, the upstream molecular mechanisms that initiate and sustain macrophage M1 polarization—particularly the communication between resident vascular cells and immune cells—remain incompletely defined.

As the principal structural component of the vessel wall, VSMCs undergo a switch from a “contractile” to a “secretory” phenotype under pathological conditions (7). Phenotypically switched VSMCs not only lose contractile function but also acquire the capacity to synthesize and secrete large amounts of cytokines and matrix proteins, thereby actively shaping the surrounding immune microenvironment. Thrombospondin-1 (THBS1) is a matricellular protein secreted by activated vascular cells and has been implicated in vascular remodeling and inflammatory responses (8, 9). CD47 is a high-affinity receptor for THBS1 and is widely expressed on immune cells. Recent studies have demonstrated that the THBS1–CD47 axis plays a critical role in regulating macrophage inflammatory responses in vascular diseases such as atherosclerosis (10, 11). Moreover, the NF-κB signaling pathway is a canonical regulator of macrophage M1 polarization, and activation of CD47 has been reported to modulate NF-κB nuclear translocation (12). Nevertheless, whether secretory VSMCs in the IA microenvironment induce macrophage M1 polarization through THBS1 release and subsequent activation of macrophage CD47/NF-κB signaling remains to be elucidated.

In this study, we sought to systematically dissect the interaction between secretory VSMCs and macrophages in IAs. By integrating bulk RNA-sequencing and single-cell RNA-sequencing data from the Gene Expression Omnibus (GEO), and applying weighted gene co-expression network analysis (WGCNA) and cell–cell communication analysis, we identified a significantly upregulated THBS1–CD47 ligand–receptor axis potentially associated with inflammatory responses in IAs. We then established an *in vitro* co-culture model and, through gene knockdown and functional rescue experiments, validated the hypothesis that secretory VSMC-derived THBS1 promotes macrophage M1 polarization and inflammatory cytokine release via the CD47/NF-κB axis. These findings may provide new potential therapeutic targets for anti-inflammatory treatment of intracranial aneurysms.

## 2 Materials and methods

### 2.1 Data acquisition

**Figure 1:**
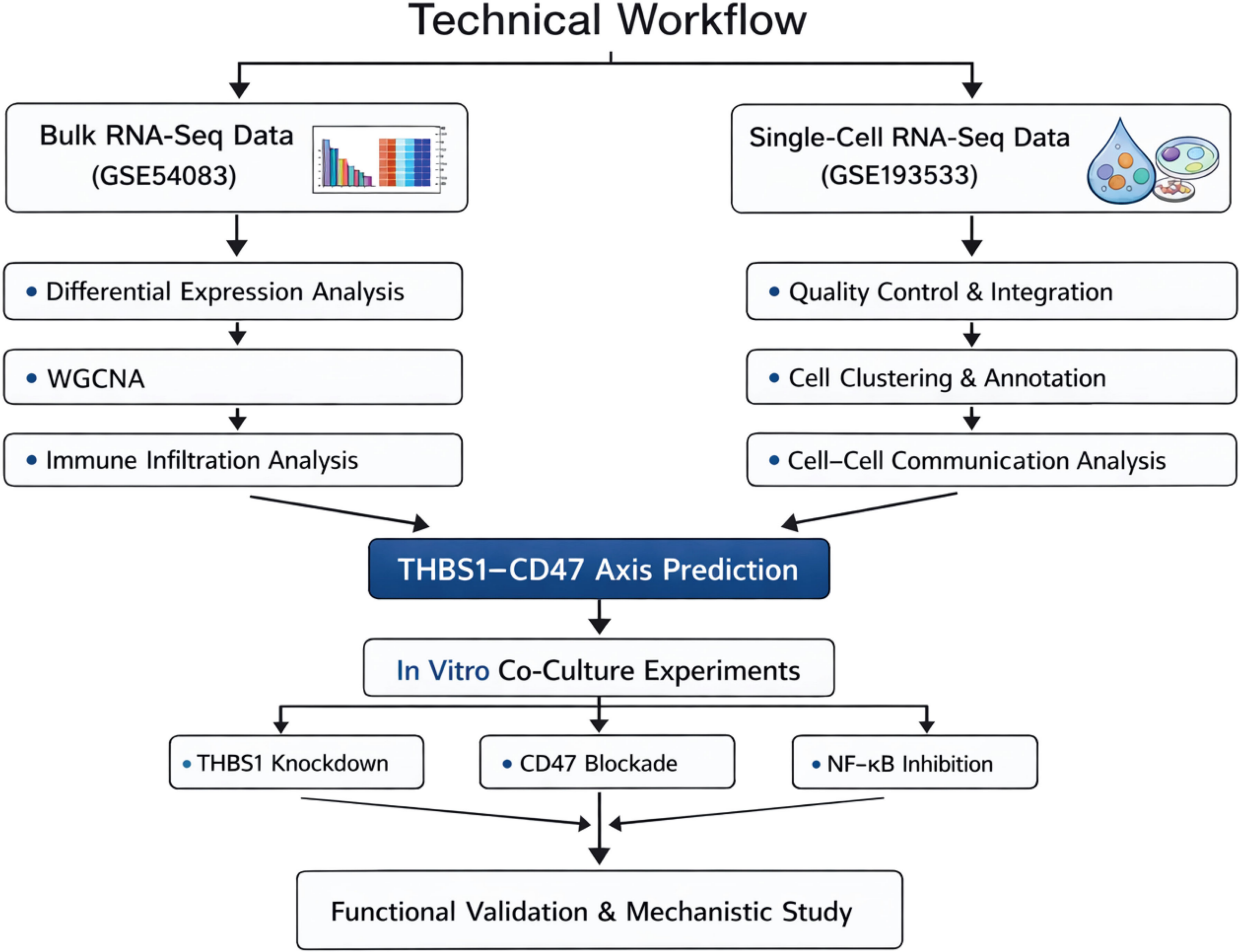
Technical roadmap of this study.

Gene expression data were obtained from the Gene Expression Omnibus (GEO) database under accession number GSE54083. This dataset is based on the GPL4133 platform and includes 15 samples (superficial temporal artery tissues and unruptured intracranial aneurysm tissues), which were divided into a Control group (n = 10) and a Test group (n = 5). The Control group comprised 10 samples and the IA group comprised 5 samples, representing the biological replicates for all subsequent analyses. Quantile normalization of all arrays was performed using the normalizeBetweenArrays function in the limma package (13) (v3.58.1) to eliminate technical variation. For multiple probes mapping to the same gene symbol, the probe with the largest row variance was retained to ensure data uniqueness and robustness. **Figure 1** illustrates the overall technical workflow of this study.

### 2.2 Differential expression analysis

Differential expression analysis was conducted using the limma workflow (13). Genes with an absolute log2 fold change (|logFC|) > 1 and an adjusted p value (adj.P.Val) < 0.05 were defined as significantly differentially expressed genes (DEGs). Quality control visualizations—including expression distribution boxplots, sample correlation heatmaps, and principal component analysis (PCA) plots—were generated to assess normalization efficiency and sample clustering.

### 2.3 Weighted gene co-expression network analysis

The WGCNA package (14) (v1.73) was used to construct weighted gene co-expression networks to identify modules of highly correlated genes and their associations with sample traits. Prior to network construction, the top 5,000 genes with the highest median absolute deviation (MAD) were selected from the normalized expression matrix to focus on the most variable transcripts. A soft-thresholding power (β) was determined based on the scale-free topology criterion, targeting a scale-free topology fit index (R^2^) > 0.85. Co-expression modules were identified using dynamic tree cutting with a minimum module size of 200 genes and a module merging threshold of 0.3. Module–trait relationships were evaluated by calculating Pearson correlations between module eigengenes and sample groups. Intramodular connectivity and module membership were used to assess the importance of individual genes within modules; hub genes were defined as those with high intramodular connectivity and high module membership (absolute correlation approaching 1).

### 2.4 Gene set enrichment analysis

To investigate the impact of high versus low expression of specific genes on global pathway activity, single-gene-based gene set enrichment analysis was performed. For each target gene, samples were divided into “high-expression” and “low-expression” groups based on the median expression across the cohort. Differential expression analysis between these two groups was then conducted using the limma package (13) (v3.58.1). Ranked gene lists were subjected to enrichment analysis against predefined “MSigDB Hallmark” gene sets using the fgsea package (15) (v1.73). Multiple hypothesis testing was corrected using the Benjamini–Hochberg method. Significantly enriched pathways (adjusted p < 0.05) were visualized using bar plots of normalized enrichment scores (NES), highlighting biological processes activated in either the high- or low-expression group.

Gene Ontology (GO) (16) and Kyoto Encyclopedia of Genes and Genomes (KEGG) (17) pathway enrichment analyses were performed on target gene sets using the clusterProfiler package (18) (v4.10.1). Enrichment significance was assessed using a hypergeometric test, with an adjusted p value (adj.p.val) < 0.05 considered significant.

### 2.5 Immune cell infiltration analysis

The CIBERSORT algorithm (19) (v0.1.0) with the LM22 signature matrix was applied to estimate the relative proportions of immune cell types in each sample. Quantile normalization mode was used, and 1,000 permutations were performed to generate p values assessing deconvolution confidence. Differences in immune cell infiltration between groups were compared using boxplots, with intergroup comparisons assessed by the non-parametric Kruskal–Wallis test. To explore potential associations between gene expression and specific immune cell infiltration levels within the microenvironment, Spearman correlation coefficients were calculated between gene expression values and CIBERSORT-estimated immune cell proportions. Scatter plots were generated using ggplot2 (20) (v4.0.1).

### 2.6 Single-cell RNA-sequencing analysis

Single-cell RNA-sequencing data were obtained from GEO under accession number GSE193533. This dataset includes three samples: one sham-operated sample, one intracranial aneurysm sample, and one ruptured intracranial aneurysm sample. Based on the aims of this study, the first two samples were selected for analysis. Independent Seurat objects were created for each sample using the CreateSeuratObject function in the Seurat package (21) (v4.4.0). Cells were retained if they met the following criteria: number of detected genes (nFeature_RNA) between 200 and 5,000, total UMI counts (nCount_RNA) between 50 and 20,000, mitochondrial gene content < 15%, and hemoglobin gene content < 0.1%. Potential doublets were identified and removed using the scDblFinder package (22) (v1.16), with a random seed set to 123 to ensure reproducibility. scDblFinder assigns a doublet score to each cell and classifies cells as “singlets” or “doublets” automatically estimating the expected doublet rate based on recovered cell numbers. It should be noted that the scRNA-seq dataset (GSE193533) contains only one control (sham-operated) sample and one unruptured IA sample. Although this limits inter-individual generalizability, this dataset currently represents one of the few publicly available high-quality paired single-cell resources for human intracranial aneurysms. The ruptured aneurysm sample was deliberately excluded to avoid the confounding effects of the massive systemic inflammatory response associated with subarachnoid hemorrhage, thereby focusing the analysis on the cellular landscape relevant to aneurysm formation and progression.

### 2.7 Data integration and batch effect correction

Merged datasets were processed using the standard Seurat workflow, including normalization, identification of highly variable features, scaling, and principal component analysis (PCA). The top 30 principal components were used for downstream dimensionality reduction. Uniform Manifold Approximation and Projection (UMAP) embeddings were generated using the RunUMAP function based on the first 30 PCs. Initial clustering was performed using the Louvain algorithm (23) via the FindNeighbors and FindClusters functions, with a resolution of 0.6. To correct batch effects arising from sample-specific technical variation, the Harmony algorithm (24) (v1.2.4) was applied with a default lambda value of 1 to balance integration strength. UMAP and t-SNE embeddings were recalculated using Harmony-corrected dimensions, and final clustering was performed at a resolution of 0.4 to obtain biologically interpretable cell populations.

### 2.8 Cell type annotation and CellChat

Cell type identification was achieved by combining unsupervised clustering with marker gene expression analysis. Differentially expressed genes were identified using the wilcoxauc function. Major cell types were annotated based on established lineage marker expression. Vascular smooth muscle cells (VSMCs) were identified by high expression of Myh11, Acta2, and Tagln (4, 25). Fibroblasts were characterized by Dcn, Col1a1, and Lum expression. Endothelial cells showed enriched expression of Pecam1, Esam, and Cdh5. Macrophages were identified by C1qa, C1qb, and Cd68 expression. T cells expressed canonical markers including Cd28, Cd3g, and Cd3d. B cells were characterized by Cd79b, Cd79a, and Ly6d. Mast cells showed specific expression of Tpsb2, Cma1, and Mcpt4. Neutrophils were identified by S100a8 and S100a9 expression. Dendritic cells were identified by Ccr7 and Cd209a, and pericytes were characterized by Kcnj8, Rgs16, and Rgs5.CellChat algorithm (26) were applied to predict key ligand–receptor interactions.

### 2.9 Construction of cell models and gene editing

All cell lines used in this study were purchased from commercial suppliers. No animal experiments or human tissue–based experiments were performed; therefore, ethics committee approval was not required.

We first established stable cellular models to mimic specific pathological conditions. Human aortic vascular smooth muscle cells (HA-VSMCs; Lonza, CC-2571) were cultured in low-serum medium containing 0.5% fetal bovine serum (FBS; Gibco) and co-stimulated with 10 ng/mL recombinant human TGF-β1 (PeproTech, AF-100-21C) and 20 ng/mL recombinant human PDGF-BB (PeproTech, AF-100-14B) for 48 h to induce a secretory/synthetic phenotype. Meanwhile, the human monocytic cell line THP-1 (ATCC, TIB-202) was treated with 100 nM phorbol 12-myristate 13-acetate (PMA; Sigma-Aldrich, P1585) for 48 h to differentiate into resting (M0) macrophages, followed by 24 h of serum starvation to eliminate residual effects. To generate a classical M1 polarization positive control with a pro-inflammatory phenotype, M0 macrophages were stimulated with 20 ng/mL interferon-γ (IFN-γ; PeproTech, AF-300-02) and 100 ng/mL lipopolysaccharide (LPS; Sigma-Aldrich, L2630) for 24 h.

To investigate the function of THBS1, we used a lentivirus-mediated knockdown approach. shRNA targeting human THBS1 (Sigma-Aldrich, TRCN0000433805) was transduced into HA-VSMCs at a multiplicity of infection (MOI) of 10. A non-targeting control shRNA (Sigma-Aldrich, SHC002) served as the negative control. During transduction, 8 μg/mL polybrene (Sigma-Aldrich, H9268) was added to enhance infection efficiency. When HA-VSMCs reached ~70% confluence, lentiviral transduction was performed as described above. At 24 h post-transduction, the medium was replaced with fresh complete medium containing 2 μg/mL puromycin (InvivoGen, ant-pr-1) for selection. Selection was continued for 7 days until all non-transduced control cells died, yielding stable knockdown cell lines. Knockdown efficiency was verified by quantitative real-time PCR (qRT-PCR) for THBS1 mRNA levels and further validated by ELISA to quantify secreted THBS1 protein concentrations in the culture supernatant. All cells were confirmed to be mycoplasma-free using the MycoAlert^TM^ mycoplasma detection kit (Lonza, LT07-318), and only passages 4–8 were used to ensure genetic stability.

### 2.10 Establishment of the transwell co-culture system and intervention strategies

To simulate paracrine communication between cells while tightly controlling variables, we established an indirect co-culture system using Transwell^®^ inserts (Corning, CLS3396). Secretory phenotype–induced VSMCs (5×10^4^ cells/well) were seeded on the inner surface of the upper insert, and M0 macrophages (1×10^5^ cells/well) were seeded in the lower chamber. Both the upper and lower chambers were filled with 500 μL serum-free medium, and all cells were serum-starved for 12 h prior to initiating co-culture. Co-culture was conducted for 48 h at 37 °C in 5% CO_2_.

The following groups were included:(1) Blank control (Con): M0 macrophages cultured alone;(2) Secretory VSMC co-culture group (Sec): VSMCs induced with TGF-β1/PDGF-BB co-cultured with M0 macrophages;(3) THBS1 knockdown group (Sec + shT): Sec VSMCs transduced with shTHBS1 co-cultured with M0 macrophages;(4) CD47 blockade group (Sec + anti-CD47): 10 μg/mL anti-human CD47 neutralizing antibody (clone B6H12; BioLegend, 323802) added to the Sec co-culture system;(5) NF-κB inhibition group (Sec + BAY): 5 μM NF-κB inhibitor BAY 11-7082 (Selleck Chemicals, S2913) added to the Sec co-culture system;(6) Positive control: M0 macrophages stimulated with LPS/IFN-γ alone.

To delineate the causal relationship along the THBS1–CD47–NF-κB axis, functional rescue experiments were performed: in the Sec + shT group, 5 μg/mL recombinant human THBS1 protein (R&D Systems, 962-TW-025/CF) was supplemented, and on this basis, 5 μM BAY 11–7082 was further applied. For all antibody treatment groups, isotype control immunoglobulin G (IgG; BioLegend, 400102) was used as a negative control.

### 2.11 Multidimensional phenotyping and mechanistic analyses

After co-culture, macrophage activation status was systematically evaluated at three levels: gene expression, functional protein secretion, and cell-surface markers.

At the gene expression level, two sets of assays were performed:(a) Conventional gene expression: Total RNA was extracted from macrophages using TRIzol^TM^ (Invitrogen, 15596018) and reverse-transcribed into cDNA with PrimeScript^TM^ RT Reagent Kit (Takara, RR037A). qRT-PCR was performed on a QuantStudio 5 real-time PCR system (Applied Biosystems, 4485694) using TB Green^®^ Premix Ex Taq^TM^ II (Takara, RR820A). mRNA levels of IL-1β, TNF-α, and inducible nitric oxide synthase (iNOS) were measured. Primer sequences are provided in **Supplementary Table 1**. Relative expression was calculated using the 2^-ΔΔCt method, with GAPDH as the internal control. Each reaction included three technical replicates.(b) Transcription factor activity: To quantify NF-κB activation in macrophages, cells were transiently co-transfected 24 h before co-culture using Lipofectamine 3000 (Invitrogen, L3000015) with 500 ng/well pNF-κB-Luc firefly luciferase reporter plasmid (Clontech, 631744) and 50 ng/well pRL-TK Renilla luciferase plasmid (Promega, E2241) as an internal control. After 48 h of co-culture, cells were lysed and luciferase activities were measured using the Dual-Luciferase^®^ Reporter Assay System (Promega, E1910) on a GloMax luminometer (Promega, E5311). Relative NF-κB transcriptional activity was expressed as the ratio of firefly to Renilla luciferase activity.

At the protein secretion level, commercially available Quantikine^®^ ELISA kits (R&D Systems, DY201 for IL-1β; DY210 for TNF-α) were used to quantify IL-1β and TNF-α concentrations in the lower-chamber supernatants following the manufacturer’s instructions. Absorbance at 450 nm was read on a microplate reader (BioTek, Synergy H1).

At the phenotypic level, harvested macrophages were washed and resuspended in PBS and incubated with a human Fc receptor blocking reagent (eBioscience, 14-9161-73) at room temperature for 15 min. Cells were then stained with PE-conjugated anti-human CD86 antibody (clone IT2.2; BioLegend, 305406) for 30 min at 4 °C. After washing, at least 10,000 events were acquired on a BD FACSCanto^TM^ II flow cytometer (BD Biosciences, 338961). Data were analyzed using FlowJo^TM^ software (v10.8; BD Biosciences, FlowJo-v10-8-1), and results were reported as the percentage of CD86-positive cells to objectively reflect M1 polarization.

### 2.12 Statistical design and data analysis

All analyses were performed in R (version 4.3.3). All experiments were independently repeated at least three times. Data were first examined for normality and homogeneity of variance. For comparisons between two groups, a two-tailed Student’s t-test or a nonparametric Mann–Whitney U test was used as appropriate. For comparisons among multiple groups, depending on data distribution, one-way ANOVA followed by Dunnett’s or Tukey’s *post hoc* multiple-comparison tests was applied, or alternatively a nonparametric Kruskal–Wallis test with Dunn’s multiple-comparison test was used. Statistical significance was defined as two-tailed P < 0.05, and significance levels were annotated in figures as *P < 0.05, **P < 0.01, ***P < 0.001.

## 3 Results

### 3.1 Differential expression analysis

**Figure 2:**
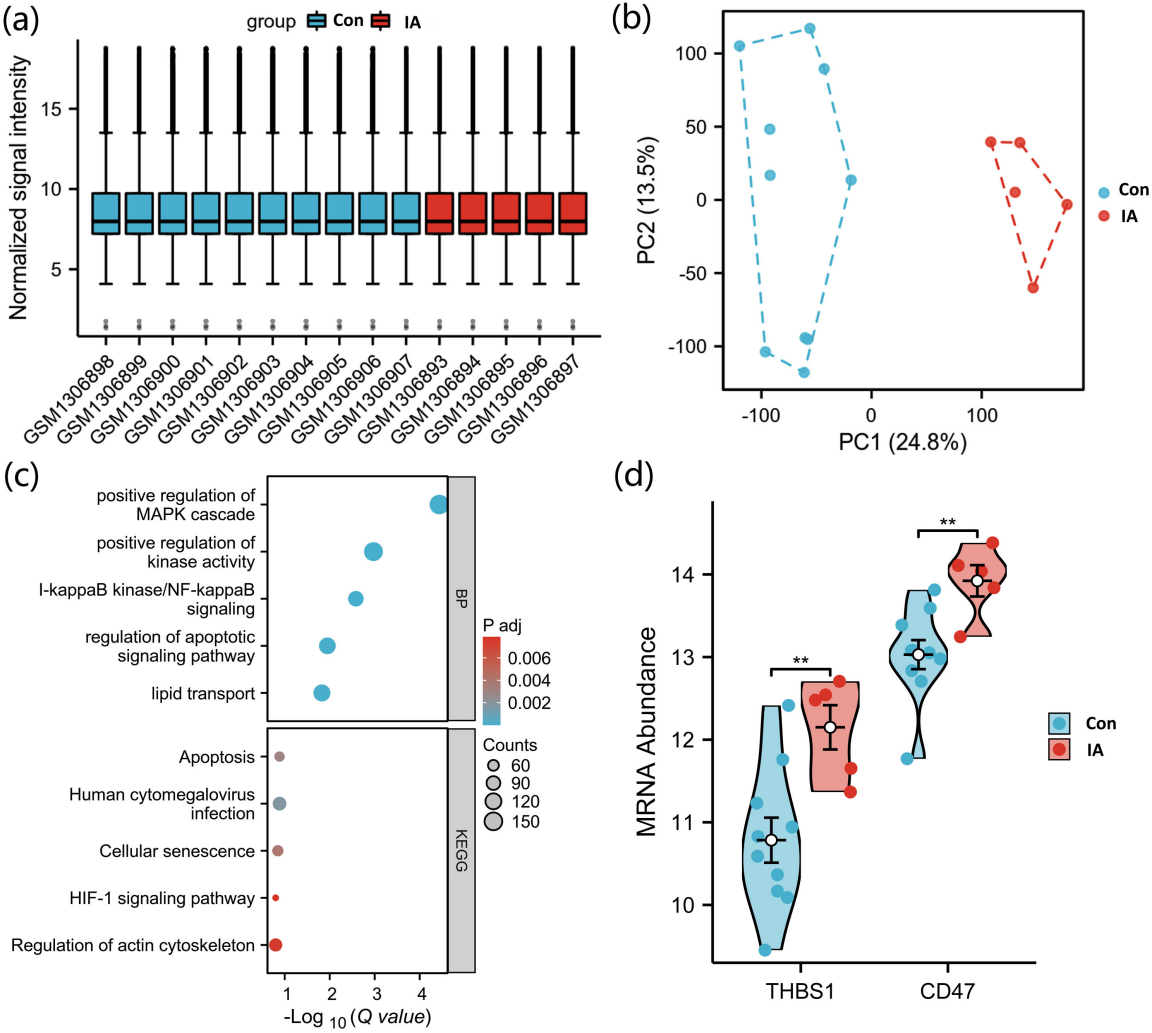
Differentially expressed genes analysis of the GSE54083 dataset. **(a)** Boxplot showing expression distribution across 15 samples (10 Control, 5 IA) after quantile normalization, with no obvious outliers detected. **(b)** PCA plot indicating clear separation between the IA and Control groups. **(c)** Bubble plot showing enriched pathways among DEGs (|log2FC| > 1, adjusted P < 0.05), including I-κB kinase/NF-κB signaling and lipid transport. **(d)** Box plots showing significant upregulation of THBS1 and CD47 in the IA group compared with the Control group. Intergroup comparisons were assessed using the Mann–Whitney U test. **P < 0.01.

All 15 samples in the GSE54083 dataset passed standard preprocessing, with no obvious outliers, and showed clear separation between the two groups (**Figures 2a, b**). Differential expression analysis identified 2,037 differentially expressed genes (DEGs), including 1,173 upregulated and 864 downregulated genes. Functional enrichment of DEGs revealed significant terms/pathways including I-kappaB kinase/NF-kappaB signaling, lipid transport, Human cytomegalovirus infection, and the HIF-1 signaling pathway (**Figure 2c**). Further analysis showed that THBS1 and CD47 were significantly upregulated in the aneurysm group (**Figure 2d**).

### 3.2 WGCNA

**Figure 3:**
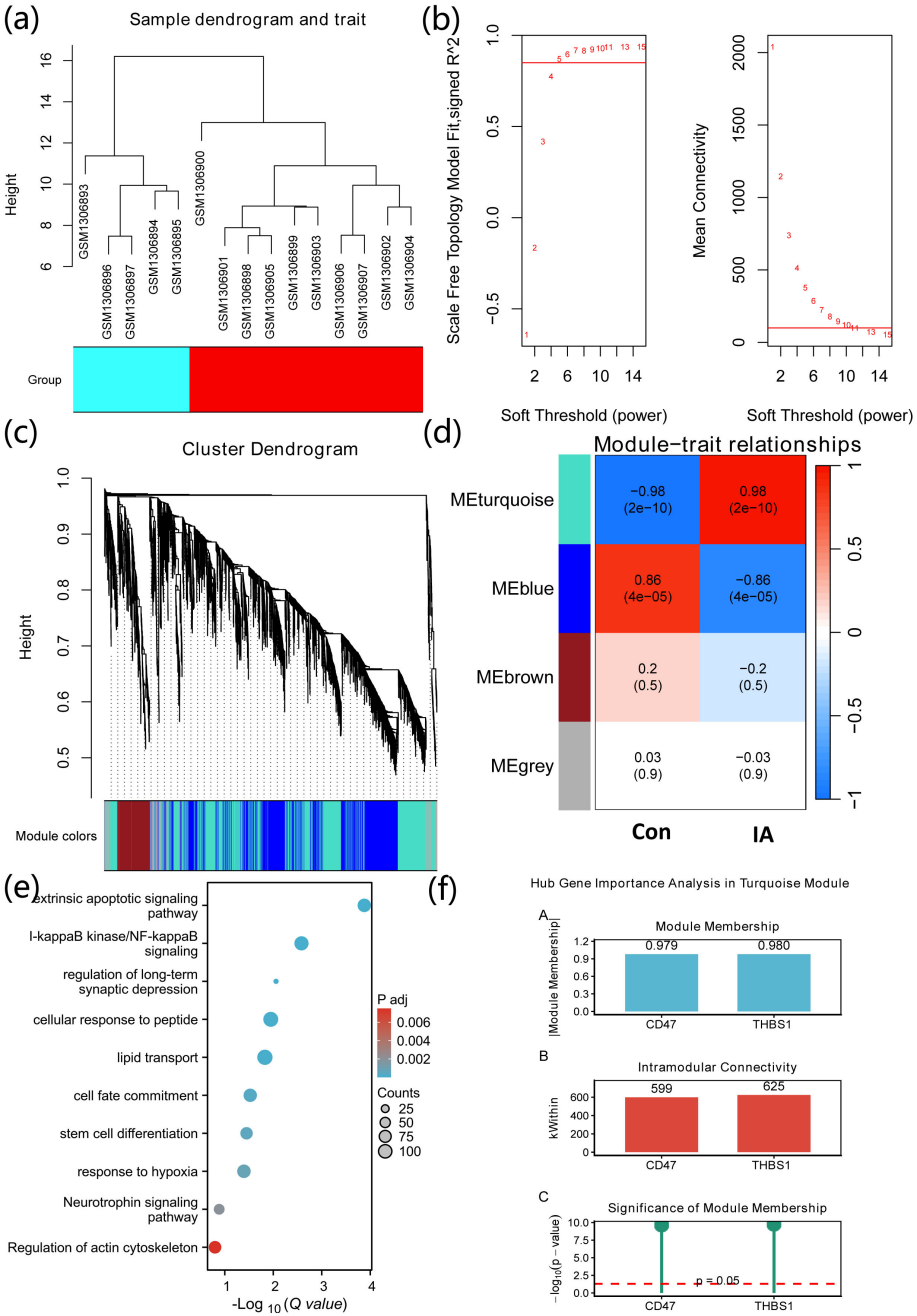
Weighted gene co-expression network analysis of the GSE54083 dataset. **(a)** Sample clustering dendrogram showing no outlier samples were removed after quality control. **(b)** Soft-thresholding power selection based on the scale-free topology criterion; optimal β = 5 with mean connectivity < 200. **(c)** Module–trait relationship heatmap showing Pearson correlations between module eigengenes and sample grouping; four co-expression modules were identified. **(d)** The turquoise module showed the strongest positive correlation with IA status (r = 0.98, p = 2×10^-10^). **(e)** GO/KEGG enrichment analysis of genes in the turquoise module, highlighting pathways including I-κB kinase/NF-κB signaling and lipid transport. Enrichment significance was assessed using a hypergeometric test with Benjamini–Hochberg correction (adjusted P < 0.05). **(f)** Bar plot showing intramodular connectivity and module membership (MM) for THBS1 and CD47; both genes exhibit high MM values (0.98 and 0.979, respectively) with MM significance p < 0.05.

After quality control, all samples were retained (**Figure 3a**). Based on the scale-free topology criterion, the optimal soft-thresholding power was set to 5, with mean connectivity < 200 (**Figure 2b**). Using dynamic tree cutting, four co-expression modules were identified, among which the turquoise module showed the strongest correlation with the group trait (r = 0.98, p = 2e-10; **Figures 3c, d**). The turquoise module contained 2,355 genes, enriched in pathways such as I-kappaB kinase/NF-kappaB signaling, lipid transport, and extrinsic apoptotic signaling pathway (**Figure 3e**). We further assessed intramodular importance of THBS1 and CD47, showing module membership values of 0.98 and 0.979, intramodular connectivity of 625 and 599, respectively, with significance of module membership < 0.05 (**Figure 3f**).

### 3.3 Immune infiltration analysis

**Figure 4:**
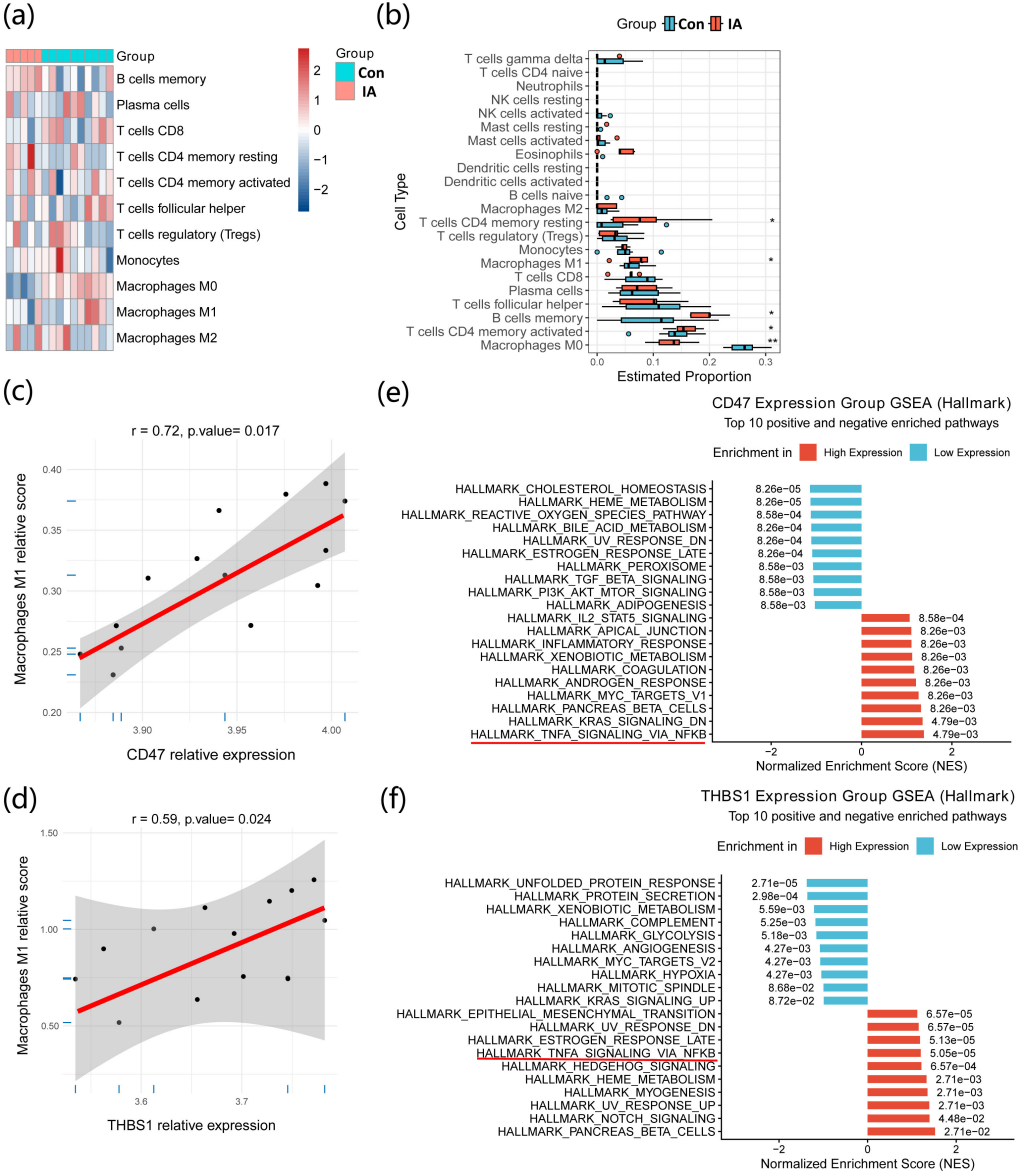
Immune infiltration analysis in intracranial aneurysm tissue and correlations with key genes. **(a)** CIBERSORT analysis showing marked differences in immune cell subsets across samples. **(b)** M1-like macrophage infiltration is significantly increased in the aneurysm group. (c–d) Spearman correlations between M1-like macrophage infiltration and CD47/THBS1 expression. **(e–f)** Single-gene GSEA linking high CD47/THBS1 expression to positive enrichment of the NF-κB signaling pathway (MSigDB Hallmark gene sets; Benjamini–Hochberg adjusted P < 0.05).

Given the enrichment results above, CIBERSORT immune infiltration analysis was performed. Immune infiltration levels varied substantially across samples (**Figure 4a**). Differential analysis showed a trend toward increased infiltration of M1-like macrophages, activated CD4 memory T cells, memory B cells, and resting CD4 memory T cells, while M0-like macrophage infiltration was significantly decreased (**Figure 4b**). Spearman correlation analysis indicated that M1-like macrophage abundance was positively correlated with CD47 (r = 0.72, p = 0.017) and THBS1 (r = 0.59, p = 0.024) expression (**Figures 4c, d**). Single-gene GSEA further suggested that high CD47 and THBS1 expression was associated with upregulation of the NF-κB signaling pathway (**Figures 4e, f**).

### 3.4 Single-cell analysis

**Figure 5:**
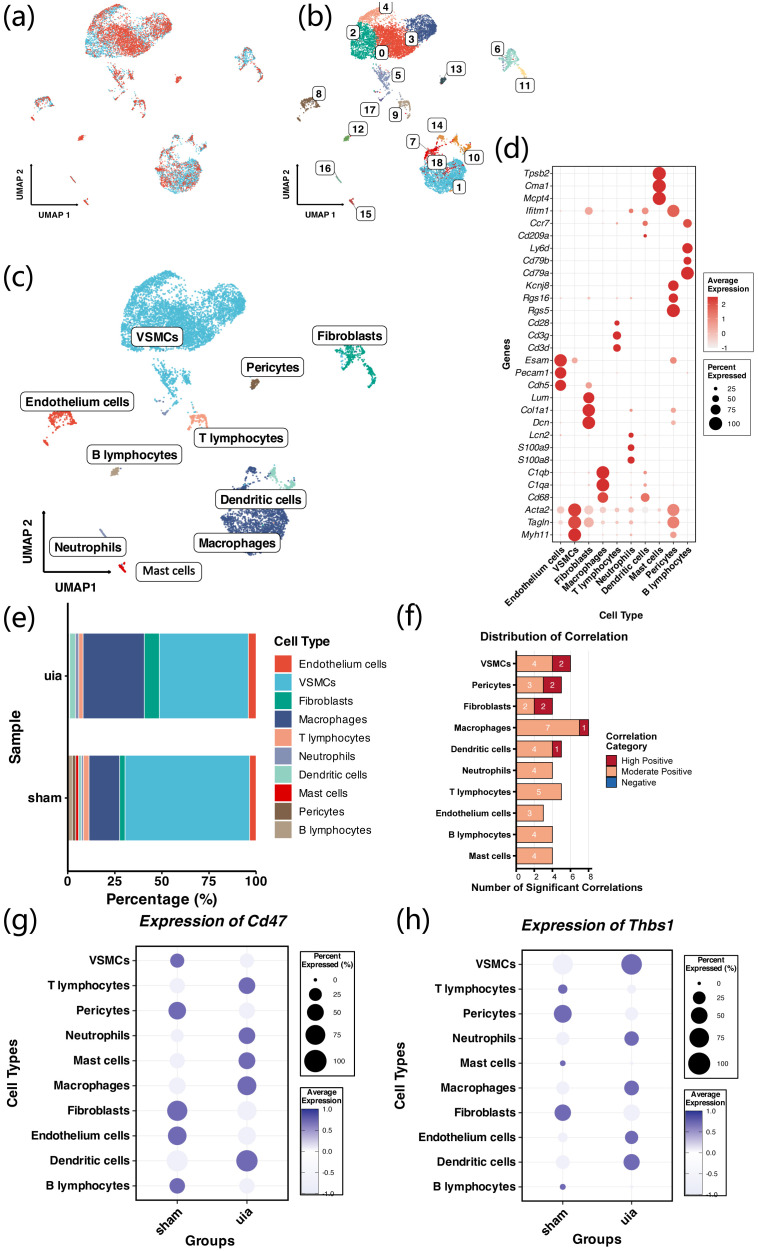
Integration, clustering, and cell-type annotation of single-cell RNA-seq data. **(a)** Harmony-corrected UMAP showing effective batch removal. **(b, c)** 18 clusters identified and annotated into 10 major cell types. **(d)** DotPlot showing cell-type-specific marker expression. **(e)** Increased M1 macrophages and fibroblasts, decreased VSMCs in aneurysm samples. **(f)** High inter-cell-type correlations (r > 0.75). **(g, h)** Differential expression of CD47 and THBS1 across groups and cell types.

After QC and doublet removal, 9,768 high-quality cells were retained. Following Harmony integration, batch effects were effectively corrected as shown by the UMAP plot (**Figure 5a**). After dimensionality reduction and clustering, cells were divided into 18 clusters (cluster0–17) and annotated into 10 major cell types (**Figures 5b, c**). DotPlot analysis demonstrated strong specificity of canonical marker genes across cell types (**Figure 5d**). In the analyzed samples, we observed a higher proportion of M1 macrophages and fibroblasts in aneurysm samples, whereas VSMCs appeared reduced. (**Figure 5e**). Correlation analysis based on average expression revealed strong correlations among M1 macrophages, fibroblasts, VSMCs, and other cell types (r > 0.75; **Figure 5f**). We further examined CD47 and THBS1 expression across groups: CD47 was significantly upregulated in dendritic cells (DCs) and macrophages in aneurysm tissue, while THBS1 was significantly upregulated in VSMCs and DCs in aneurysm tissue (**Figures 5g, h**). These findings suggest that the THBS1–CD47 axis may mediate interactions between VSMCs and macrophages.

### 3.5 Secondary analysis of macrophages

**Figure 6:**
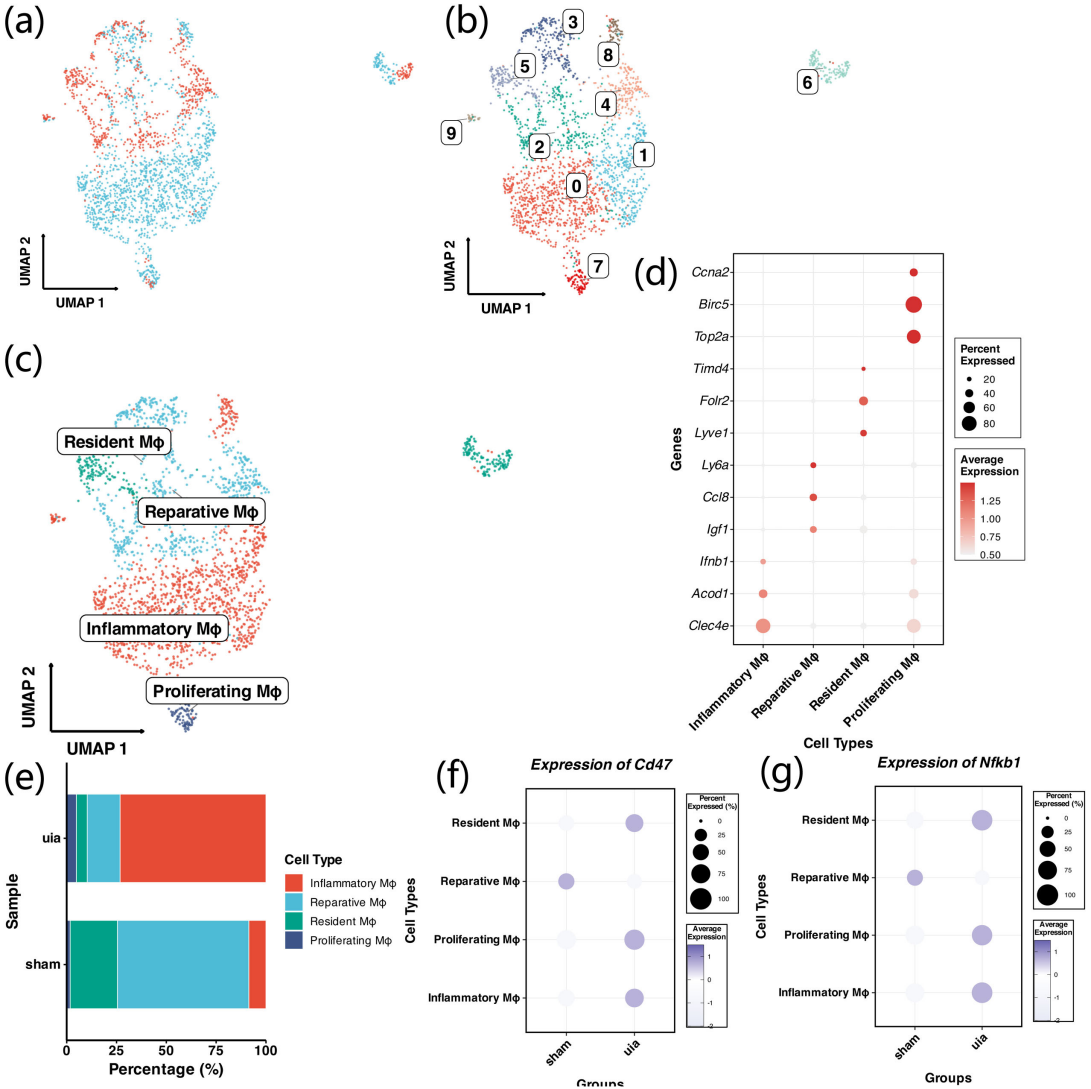
Secondary analysis and subtype identification of macrophages. **(a)** UMAP plot after Harmony integration of macrophages extracted from the full dataset, showing effective batch correction between Sham and UIA samples. **(b, c)** UMAP plots displaying 10 macrophage subclusters annotated into four major subtypes: inflammatory, reparative, resident, and proliferative macrophages. **(d)** DotPlot showing subtype-specific expression of canonical marker genes. **(e)** Bar chart comparing macrophage subtype proportions between Sham and UIA samples; inflammatory macrophages are markedly increased, whereas reparative and resident macrophages are decreased in UIA tissue. **(f, g)** Violin plots showing significant upregulation of Cd47 and Nfkb1 expression in inflammatory, proliferative, and resident macrophage subtypes in UIA compared with Sham tissue.

Macrophages were extracted from the full single-cell dataset for secondary integration, showing effective batch correction between groups (**Figure 6a**). A total of 2,414 macrophages were clustered into 10 subclusters (cluster0–9) and annotated into four macrophage types: inflammatory macrophages, reparative macrophages, resident macrophages, and proliferative macrophages (**Figures 6b, c**). DotPlot analysis showed subtype-specific expression of marker genes (**Figure 6d**). In aneurysm tissue, inflammatory macrophages were markedly increased, whereas reparative and resident macrophages were decreased (**Figure 6e**). Comparative analysis showed that Cd47 and Nfkb1 expression was significantly upregulated in inflammatory, proliferative, and resident macrophages in aneurysm tissue (**Figures 6f, g**).

### 3.6 Secondary analysis and subtype identification of VSMCs

**Figure 7:**
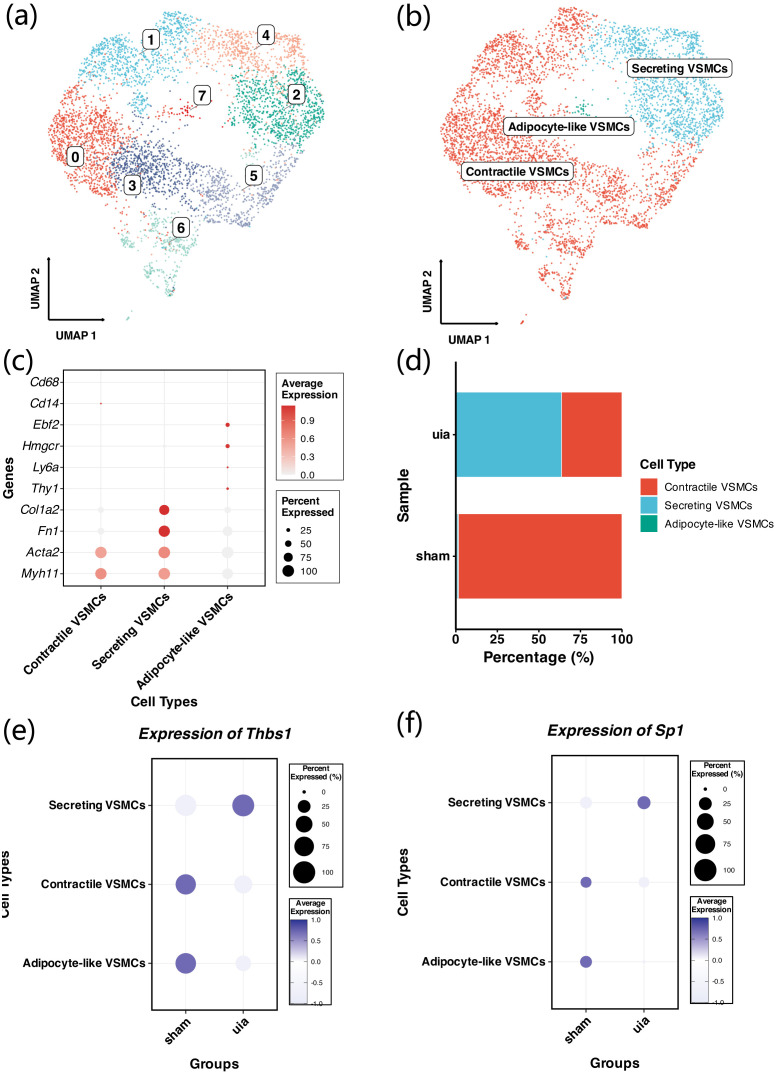
Secondary analysis and subtype identification of vascular smooth muscle cells. **(a, b)** Secondary analysis of VSMCs; 8 clusters identified and annotated into 3 VSMC subtypes. **(c)** DotPlot of canonical VSMC markers. **(d)** Increased secretory VSMCs and decreased contractile VSMCs in the aneurysm group. **(e, f)** Upregulation of Sp1 and Thbs1 in secretory VSMCs in aneurysm tissue.

VSMCs were extracted from the full dataset for secondary integration. A total of 5,500 VSMCs were clustered into 8 subclusters (cluster0–7) and annotated into three VSMC subtypes: secretory VSMCs, contractile VSMCs, and adipocyte-like VSMCs (**Figures 7a, b**). DotPlot analysis showed subtype-specific expression patterns of marker genes (**Figure 7c**). We observed a significantly higher proportion of secretory VSMCs and a lower proportion of contractile VSMCs in aneurysm tissue (**Figure 7d**). Comparative analysis showed that Sp1 and Thbs1 expression was significantly upregulated in secretory VSMCs in aneurysm tissue (**Figures 7e, f**).

### 3.7 Cell–cell communication

**Figure 8:**
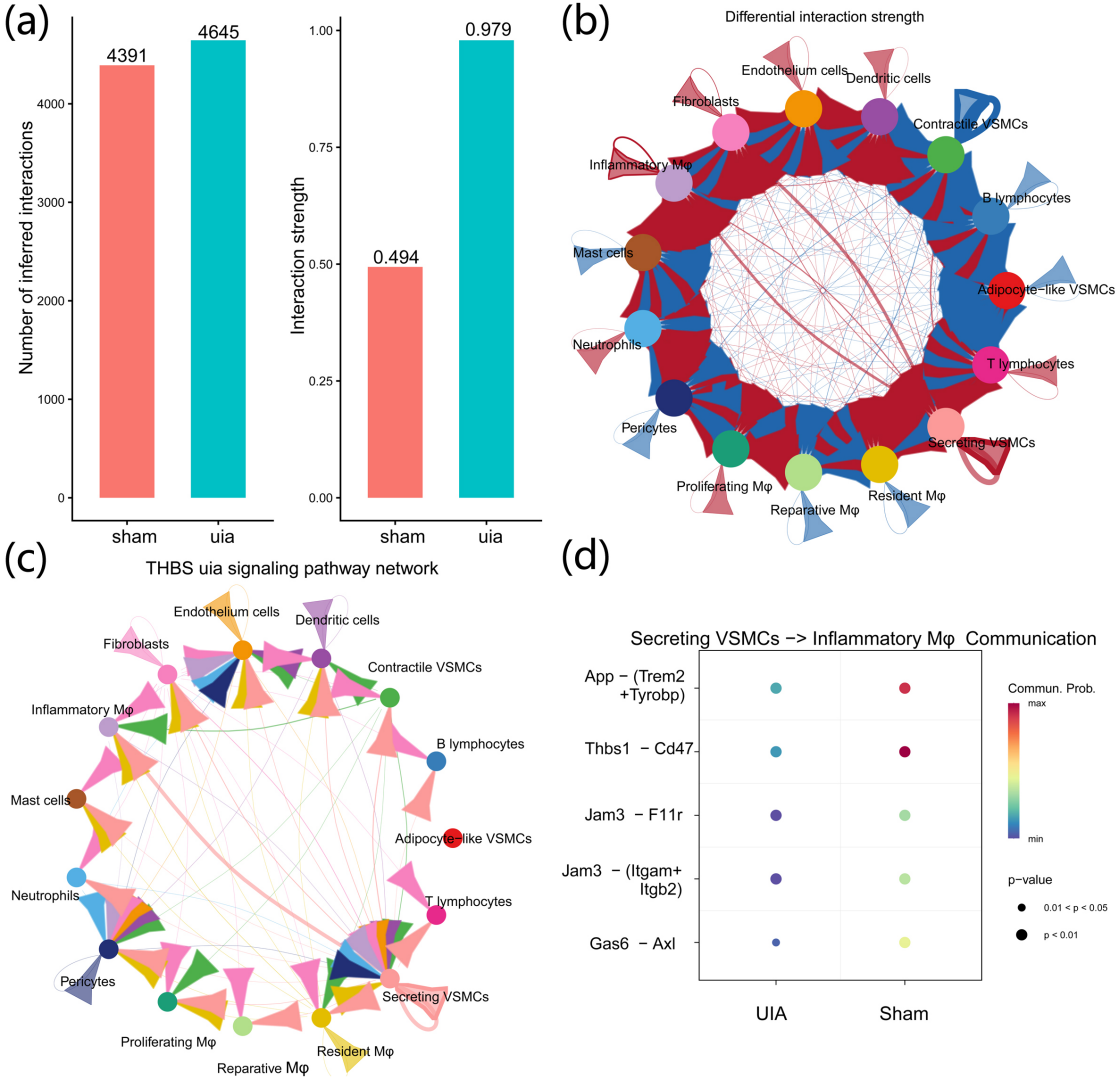
Intercellular communication analysis in intracranial aneurysm tissue. **(a)** CellChat indicates increased number and strength of communications in aneurysm samples. **(b)** Enhanced interactions of secretory VSMCs with inflammatory macrophages and fibroblasts. **(c, d)** Significantly increased communication strength via the Thbs1–CD47 axis between secretory VSMCs (sender) and inflammatory macrophages (receiver) in aneurysm samples. All ligand–receptor interactions shown represent computational predictions based on transcriptional co-expression and should be interpreted as hypothesis-generating rather than direct evidence of functional protein–protein interactions.

CellChat analysis of the full single-cell dataset inferred an overall increase in the number and strength of intercellular communications in aneurysm tissue (**Figure 8a**). The predicted interaction strength between secretory VSMCs and inflammatory macrophages as well as fibroblasts was notably increased in the aneurysm group (**Figure 8b**). Further analysis predicted that, in aneurysm tissue, secretory VSMCs showed high communication probability with inflammatory macrophages through the Thbs1–Cd47 ligand–receptor axis (**Figures 8c, d**). These findings represent computational inferences based on transcriptional co-expression and should be interpreted as hypothesis-generating rather than direct evidence of functional protein–protein interactions.

### 3.8 Secretory VSMCs promote macrophage M1 polarization and inflammatory activation via the THBS1/CD47/NF-κB axis

**Figure 9:**
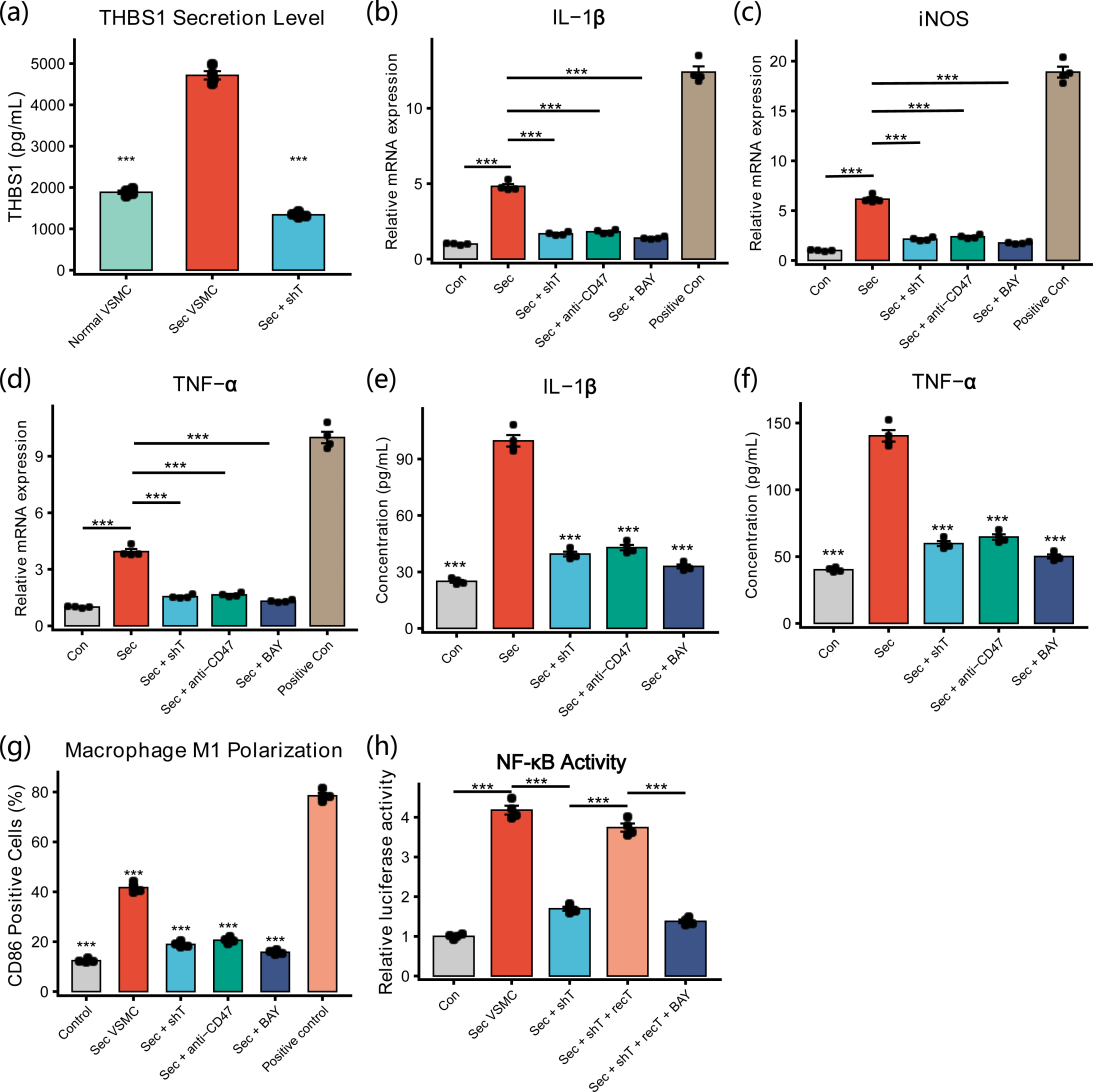
Secretory VSMCs promote macrophage M1 polarization and inflammatory activation through the THBS1/CD47/NF-κB axis. **(A)** ELISA quantification of THBS1 secretion from HA-VSMCs before and after TGF-β1/PDGF-BB stimulation and following shTHBS1 transduction. **(B–D)** qRT-PCR analysis of macrophage IL-1β, TNF-α, and iNOS mRNA in the Transwell co-culture system. **(E, F)** ELISA quantification of IL-1β and TNF-α secretion in co-culture supernatants. **(G, H)** Flow cytometric analysis of CD86^+^ macrophages and NF-κB transcriptional activity (Dual-luciferase reporter assay). All *in vitro* experiments were independently performed in three biological replicates (n = 3). Data are presented as mean ± standard deviation (SD). Statistical comparisons among multiple groups were performed using one-way analysis of variance (ANOVA) followed by Tukey’s *post hoc* multiple-comparison test. *P < 0.05, **P < 0.01, ***P < 0.001.

First, THBS1 secretion from HA-VSMCs stimulated with TGF-β1/PDGF-BB was significantly increased (4715 ± 199 pg/mL vs. 1885 ± 81 pg/mL in unstimulated VSMCs, P < 0.001), and was reduced to 1340 ± 59 pg/mL after shTHBS1 transduction, corresponding to an approximate knockdown efficiency of 72% (P < 0.001; **Figure 9a**). Second, Transwell co-culture experiments showed that, compared with co-culture with unstimulated VSMCs, secretory VSMCs significantly upregulated macrophage mRNA expression of IL-1β (4.82 ± 0.31-fold), TNF-α (3.94 ± 0.27-fold), and iNOS (6.15 ± 0.39-fold). These pro-inflammatory genes were significantly downregulated following THBS1 knockdown, CD47 neutralization (anti-CD47), or NF-κB inhibition by BAY 11-7082 (all P < 0.001; **Figures 9b–d**). Consistently, secretion levels of IL-1β and TNF-α in the supernatants were significantly increased and were markedly reduced by shTHBS1, anti-CD47, or BAY11–7082 treatment (P < 0.001; **Figures 9e, f**). Flow cytometry revealed a similar pattern for the proportion of CD86^+^ cells, an M1 marker (P < 0.001; **Figure 9g**).

Dual-luciferase reporter assays further showed that co-culture with secretory VSMCs robustly activated macrophage NF-κB transcriptional activity (4.18 ± 0.22-fold, P < 0.001), which was significantly decreased after THBS1 knockdown (1.70 ± 0.11-fold, P < 0.001). Supplementation with recombinant THBS1 successfully rescued this effect (restored to 3.74 ± 0.19-fold, P < 0.001), whereas combined treatment with BAY 11–7082 again significantly suppressed NF-κB activity (1.38 ± 0.08-fold, P < 0.001; **Figure 9h**), supporting the hypothesis that THBS1 mediates macrophage inflammatory activation through the CD47/NF-κB pathway.

## 4 Discussion

By integrating multi-omics bioinformatics analyses with *in vitro* functional experiments, this study explored potential communication mechanisms between vascular smooth muscle cells (VSMCs) and macrophages in the microenvironment of intracranial aneurysm (IA). The major findings are as follows: (1) combined transcriptomic and single-cell analyses indicated patterns consistent with VSMC phenotypic switching and marked infiltration of M1 macrophages in IA tissue; (2) CellChat-based ligand–receptor analysis predicted that the THBS1–CD47 axis could be a key signaling pathway linking secretory VSMCs and inflammatory macrophages; and (3) *in vitro* cellular models confirmed that, under simulated pathological conditions, THBS1 derived from secretory VSMCs induces macrophage polarization toward a pro-inflammatory phenotype through activation of the CD47/NF-κB signaling axis. Together, these findings provide a new perspective for understanding chronic inflammation within the IA vessel wall.

Inflammatory responses and extracellular matrix degradation are considered core pathophysiological processes underlying IA formation and rupture (3, 27). In our analysis of the single-cell dataset, we observed a trend toward increased proportions of pro-inflammatory M1-like macrophages in aneurysm samples compared with controls, consistent with previous reports emphasizing macrophages as a major driver of IA progression (3, 28, 29). Although the sample size of the single-cell dataset was limited and may be influenced by inter-individual heterogeneity, it is noteworthy that our immune infiltration analysis based on a larger bulk RNA-seq cohort produced highly concordant results. This cross-platform consistency strengthens the reliability of our conclusion that immune microenvironment remodeling is a common feature of IA. In addition, our analyses captured features of VSMC transition from a “contractile” to a “secretory/synthetic” phenotype, characterized by decreased expression of contractile proteins (e.g., ACTA2) and increased expression of genes involved in biosynthesis and metabolism. Prior studies have indicated that phenotypically switched VSMCs lose their vessel tone–maintaining function and instead become active regulators of the local microenvironment (30–33).

A key finding of this study is the identification of the THBS1–CD47 axis as a potentially important mediator of communication between secretory VSMCs and macrophages. THBS1 is a stress-inducible matricellular protein with dual roles in vascular injury repair. While plasma THBS1 levels have been associated with abdominal aortic aneurysm expansion (34), the cellular source and local mechanistic roles of THBS1 in IA remain unclear. Our bioinformatics analyses suggested that upregulated THBS1 in IA tissue likely originates from secretory VSMCs, whereas its receptor CD47 is mainly expressed in immune cells. This suggests that VSMCs may paracrinally release THBS1 as a danger-associated molecular pattern (DAMP) to actively modulate immune cell function, which is consistent with findings reported by Liu et al. in an abdominal aortic aneurysm model (35).Furthermore, THBS1–CD47 signaling has been shown to regulate downstream inflammatory programs in vascular and immune contexts beyond its classical anti-phagocytic role (36). It is important to emphasize that the ligand–receptor interactions inferred by CellChat are based on transcriptional co-expression and probabilistic models, and do not in themselves constitute evidence of physical protein binding or downstream signaling activation. Ideally, spatial validation strategies—such as dual immunofluorescence or multiplex immunohistochemistry for THBS1–CD47 co-localization in human aneurysm tissue, or spatial transcriptomics to confirm ligand–receptor proximity at the tissue level—would provide more direct evidence for this intercellular communication. However, obtaining sufficient structurally intact unruptured aneurysm wall specimens for high-quality histological and spatial analyses is technically and ethically challenging at our center, given the increasing clinical preference for endovascular coiling over open surgical clipping. We have explicitly listed the absence of tissue-level protein validation as a limitation of this study and designated it as a key priority for future specimen collection efforts.

Mechanistically, our *in vitro* co-culture experiments provide causal evidence supporting the bioinformatic predictions. We found that blocking CD47 or knocking down THBS1 in VSMCs significantly suppressed co-culture–induced release of inflammatory cytokines (IL-1β and TNF-α) from macrophages. Importantly, we demonstrated that this process depends on activation of the NF-κB pathway. Although CD47 is commonly recognized as an anti-phagocytic “don’t eat me” signal, emerging immunological evidence indicates that CD47 activation can promote inflammatory signaling in non-tumor settings. For example, Gao et al. reported that CD47 activation facilitates IκBα degradation, thereby enabling NF-κB nuclear translocation (12). Our study proposes this mechanism to the IA context and, through rescue assays, provides evidence supporting an chain of “VSMC phenotypic switching → THBS1 secretion → macrophage CD47 activation → NF-κB nuclear translocation → M1 polarization”. We also acknowledge that BAY 11-7082, as a chemical inhibitor of IκBα phosphorylation, carries the potential for off-target effects that may confound mechanistic interpretation when used in isolation. To mitigate this concern, NF-κB activation in the present study was assessed primarily using a dual-luciferase reporter system (pNF-κB-Luc), which provides a functional readout of NF-κB transcriptional activity independent of the inhibitor. The convergence of multiple orthogonal readouts—reporter activity, cytokine secretion (IL-1β, TNF-α by ELISA), surface marker expression (CD86 by flow cytometry), and pro-inflammatory gene transcription (iNOS by qRT-PCR)—collectively supports NF-κB–dependent inflammatory activation. Nevertheless, we recognize that direct protein-level evidence of NF-κB activation, such as immunofluorescence detection of p65 nuclear translocation or Western blot analysis of IκBα phosphorylation and degradation, would further strengthen the mechanistic conclusion. These experiments are planned as part of our ongoing follow-up studies.

Furthermore, the interpretation of CD47 function requires a nuanced understanding of its context-dependent signaling, which differs significantly between neoplastic and non-neoplastic vascular environments. Classically, in the field of immuno-oncology, CD47 is well-characterized as a “don’t eat me” signal that interacts with SIRPα on macrophages to inhibit phagocytosis, thereby allowing tumor immune evasion (37, 38). However, in the context of vascular inflammation and sterile injury, accumulating evidence suggests that CD47 operates via distinct mechanisms (10). Unlike the inhibitory SIRPα pathway, the ligation of CD47 by matricellular proteins such as THBS1 can trigger active pro-inflammatory signaling cascades. Specifically, previous studies have demonstrated that THBS1–CD47 interaction promotes the phosphorylation of downstream effectors, leading to the ubiquitination and degradation of IκBα, which in turn facilitates the nuclear translocation of NF-κB (39, 40). This mechanistic distinction explains why, in our aneurysm model, the blockade of the THBS1–CD47 axis resulted in a marked suppression of NF-κB–dependent cytokine production (IL-1β and TNF-α) and M1 polarization, rather than merely altering macrophage phagocytic activity. Thus, our findings support the view that in the IA microenvironment, CD47 functions primarily as a pro-inflammatory receptor driving the “cytokine storm” and vessel wall remodeling, distinct from its immune-checkpoint role in cancer.

Several limitations of this study should be acknowledged. First, the bulk transcriptomic dataset (n = 15) and scRNA-seq dataset (n = 2, effectively 1-versus-1 after exclusion of the ruptured sample) are both limited in size. While comparable sample sizes are common in the intracranial aneurysm field given the ethical constraints and scarcity of surgical specimens, the small cohort increases the risk of overfitting in WGCNA and limits inter-individual generalizability. We addressed this partially by employing dynamic tree cutting (minimum module size = 200 genes), which yielded high module stability (turquoise module: r = 0.98 with group trait, p = 2×10^-10^; THBS1 and CD47 module membership = 0.98 and 0.979, respectively). Leave-one-out validation was not feasible given insufficient per-iteration statistical power, and external dataset integration (e.g., GSE15629, GSE36791) was precluded by cross-platform heterogeneity; both are designated as priorities for future work. It should also be noted that CIBERSORT-based immune deconvolution carries inherent limitations in solid tissues, as peripheral blood–derived signature matrices may incompletely capture tissue-resident immune cell states (41, 42), further underscoring the exploratory nature of our immune infiltration estimates. The scRNA-seq findings should therefore be regarded as hypothesis-generating, which is precisely why we adopted a three-tiered validation strategy—bulk RNA-seq, single-cell profiling, and *in vitro* functional experiments—to compensate for the limitations of any single dataset. Second, control samples were derived from superficial temporal artery (STA) tissue rather than normal intracranial arterial tissue, which is ethically unobtainable from living patients. Although STA is the most widely accepted surrogate control in this field—as reflected by its use in the original GSE54083 study published in Stroke—inherent biological differences in vessel caliber, wall composition, hemodynamic shear stress, and embryonic origin (mesodermal STA vs. neural crest–derived intracranial vessels) mean that some observed transcriptomic differences may reflect tissue-type variation rather than aneurysm-specific pathology. Our conclusions should be interpreted accordingly. Third, mechanistic experiments relied on human aortic HA-VSMCs and THP-1–derived macrophages rather than primary intracranial aneurysm cells, which are ethically and technically difficult to obtain in sufficient quantities. The mesodermal origin of HA-VSMCs differs from the neural crest origin of intracranial VSMCs, and transcriptional responses to pathological stimuli may not be fully equivalent between these populations. Nevertheless, VSMC phenotypic switching and the THBS1–CD47–NF-κB axis represent conserved vascular biological processes, and our cell line system provides a reproducible platform for delineating specific molecular causality. Finally, NF-κB activation was assessed primarily via a dual-luciferase reporter system rather than direct protein-level readouts (e.g., p65 nuclear translocation or IκBα degradation by Western blot); although the convergence of multiple functional readouts (cytokine secretion, CD86 expression, iNOS transcription) collectively supports our conclusions, these additional mechanistic validations and future *in vivo* aneurysm model studies remain important directions.

## 5 Conclusion

In summary, by integrating single-cell transcriptomic profiling with *in vitro* mechanistic validation, this study highlights a potential intercellular communication pathway in the intracranial aneurysm microenvironment. Our results indicate that phenotypically switched secretory VSMCs may represent a key cellular source of thrombospondin-1 (THBS1). Secreted THBS1 acts as a paracrine signal that activates CD47 on macrophages and downstream NF-κB signaling, thereby promoting macrophage polarization toward a pro-inflammatory M1 phenotype. These findings suggest a molecular link between vessel wall remodeling and immune inflammatory responses and suggest that blocking the THBS1–CD47 signaling axis may offer a potential therapeutic strategy to suppress vessel wall inflammation and stabilize aneurysm progression.

## Data Availability

GSE54083 dataset: https://www.ncbi.nlm.nih.gov/geo/query/acc.cgi?acc=gse54083.GSE193533 dataset: https://www.ncbi.nlm.nih.gov/geo/query/acc.cgi?acc=gse193533.
